# Relative power: Explaining the effects of food and cash transfers on allocative behaviour in rural Nepalese households

**DOI:** 10.1016/j.jdeveco.2021.102784

**Published:** 2021-11-11

**Authors:** Helen Harris-Fry, Naomi M. Saville, Puskar Paudel, Dharma S. Manandhar, Mario Cortina-Borja, Jolene Skordis

**Affiliations:** a Department of Population Health, London School of Hygiene & Tropical Medicine, Keppel Street, London, WC1E 7HT, UK; b UCL Institute for Global Health, 30 Guilford Street, London, WC1N 1EH, UK; c Mother and Infant Research Activities, PO Box 921, Thapathali, Kathmandu, Nepal; d Population, Policy and Practice Research and Teaching Department, University College London Great Ormond Street Institute of Child Health, 30 Guilford Street, London, WC1N 1EH, UK

**Keywords:** Nepal, Bargaining power, Intra-household food allocation, Diet, Equity

## Abstract

We estimate the effects of antenatal food and cash transfers with women’s groups on household allocative behaviour and explore whether these effects are explained by intergenerational bargaining among women. Interventions were tested in randomised-controlled trial in rural Nepal, in a food-insecure context where pregnant women are allocated the least adequate diets. We show households enrolled in a cash transfer intervention allocated pregnant women with 2–3 pp larger shares of multiple foods (versus their mothers-in-law and male household heads) than households in a control group. Households in a food transfer intervention only increased pregnant women’s allocation of staple foods (by 2 pp). Intergenerational bargaining power may partly mediate the effects of the cash transfers but not food transfers, whereas household food budget and nutrition knowledge do not mediate any effects. Our findings highlight the role of intergenerational bargaining in determining the effectiveness of interventions aiming to reach and/or empower junior women.

## 1 Introduction

Food allocation in South Asian households is notably more biased against women than in other parts of the world (Akerele, 2011; Berti, 2012; Calvi, 2020; Coates et al., 2017),^1^ yet women are often responsible for these allocation decisions. In patrilocal-patrilineal South Asian societies, where daughters relocate to their husband’s parental home after marriage, the power dynamics between spouses and between daughters-in-law and mothers-in-law may influence the allocation of food (Agarwal, 1994; Kandiyoti, 1988; Morrison et al., 2017). These allocative choices are important in this context, where food shortages are common and the prevalence of undernutrition in women and children are among the highest in the world (Global Nutrition Report, 2020).

Several studies have documented effects of gendered bargaining power – that is, women’s versus men’s ability to influence household decisions – on household-level consumption and expenditures (e.g., Attanasio and Lechene (2014); Hoddinott and Haddad (1995); Quisumbing and de La Brière (2000)). These studies find widely differing effects of gendered bargaining power on the shares of household budget spent on different goods. There is less evidence on the effects on food allocation to different household members, although women’s bargaining power has been positively associated with women’s food shares and dietary diversity in Bangladesh (D’Souza and Tandon, 2019; Rahman, 2012; Sraboni and Quisumbing, 2018), maternal dietary diversity and body-mass index in Nepal (Malapit et al., 2015), and better health outcomes in India (Calvi, 2020).

A large anthropological literature suggests that intergenerational bargaining among women also determines intra-household allocations of food (Bennett, 1983; Cornwall, 2007; Vera-Sanso, 1999). In fact, intergenerational bargaining power may be a stronger determinant in some contexts. This may be particularly true where mothers-in-law control everyday food purchasing, preparation, and distribution decisions in joint households, and men tend to control larger expenditures (Aubel, 2012; Morrison et al., 2017). Relationships between mothers-in-law and daughters-in-law are complex: women may compete for their husband/son’s affections whilst also feeling a duty of care to one another (Gram et al., 2018; Kandiyoti, 1988). This relationship is further complicated when daughters-in-law are pregnant and carrying their mother-in-law’s grandchild (Aubel, 2012). Beyond its physiological importance, food allocation can be a nurturing, social act of commensality, whilst withholding or refusing food can communicate disrespect, discontent, or punishment (Harriss-White et al., 1991).

In South Asia, these intergenerational power dynamics are changing, as divorce remains rare but division from joint into nuclear households is increasingly common, strengthening the outside options for daughters-in-law vis-á-vis their mothers-in-law (Vera-Sanso, 1999). Increasing male outmigration for work also changes these dynamics, resulting in more female-only households and, in some cases, overseas remittances being secretly saved to facilitate household separation (Gram et al., 2018). It has recently been shown that an Indian woman’s co-residence with her mother-in-law constrains her social connections, in turn reducing her access to modern family planning (Anukriti et al., 2020), and that a larger network of ‘in-laws’ in Nepal constrains women’s ability to act on acquired health knowledge (Skordis et al., 2019). Intergenerational bargaining effects on intra-household resource allocation are under-researched, although D’Souza and Tandon (2019) find that the presence of a mother-in-law in Bangladeshi households increases the equity of food distribution, by allocating herself (the mother-in-law) more food. Calvi (2020) finds that the bargaining power of Indian women (aged 15–80 years) and their allocation of non-food resources, has an inverted U-shaped relationship with age.

It is surprising, therefore, that most nutrition, health, and social welfare interventions overlook these intergenerational power dynamics in both design and evaluation. Nutrition interventions usually recognise and may even reinforce women’s traditional role in food preparation and allocation, for example by selectively providing women with food, other resources, or nutrition education. Some intervention studies have also shown that women’s empowerment can partially mediate intervention effects on health outcomes, for example in studies on the effects of agricultural interventions or cash transfers (Heckert et al., 2019; Tommasi, 2019). However, studies rarely consider the gatekeeping role that older women such as mothers-in-law can play in determining intervention success (Concha and Grandmothers, 2021). This may be because most economic models of household behaviour conceptualise household allocation as a function of preferences of a single dictator as in Becker’s unitary model (Becker, 1981), or of men and women as is the case with most applications of the collective model (Bourguignon and Chiappori, 1992). These models overlook intergenerational effects that could explain the allocation of resources across both gender and generations in a way that may mediate an intervention’s impact.

In this paper, we report results from a cluster-randomised controlled trial testing the effects of antenatal food and cash transfers on the allocation of food in joint households in rural Nepal (protocol in Saville et al. (2016)). Pregnant women living in clusters allocated to the cash arm were eligible to receive ~7.5 USD/month, and pregnant women living in clusters allocated to the food arm were eligible to receive 10 kg/month of a fortified blend of flour, soya, and sugar, called ‘Super Cereal’. Transfers were provided unconditionally to pregnant women at ‘Participatory Learning and Action’ (PLA) women’s groups. Here, we estimate the effects of the food and cash interventions on intra-household food allocation, and then explore whether these effects are explained by gains in: (1) relative or absolute bargaining power of pregnant women, (2) household budgets, or (3) nutrition knowledge and preferences.

Using dietary intake data on pregnant women, their mothers-in-law, and male household heads, we find that most people’s diets are highly deficient in macro- and micronutrients. We also find a clear gender bias in the intra-household allocation of food that favours men. This bias extends beyond differences in requirements caused by physiological sex differences and physical activity levels. Despite the increased nutritional demands of pregnancy, mothers-in-law and pregnant daughters-in-law receive similar shares of food, resulting in daughters-in-law having the lowest nutritional adequacy.

Our intention-to-treat estimates show that households in the cash intervention gave daughters-in-law larger shares of multiple foods, whereas households in the food intervention only altered their allocations of staple foods. Relative to the comparison group, households in the cash intervention allocated daughters-in-law with 2 percentage points larger shares of staple foods vs. their mothers-in-law, 2 pp larger shares of fruits and vegetables vs. their mothers-in-law, and 3 pp larger shares of animal-source foods vs. male household heads. On the other hand, the food intervention only affected the allocation of staples foods between daughters-in-law and mothers-in-law, by 2 pp.

Further analyses suggest that these differences in treatment effects are partially explained by differing effects on bargaining power. The cash intervention had a modest effect on the bargaining power of daughters-in-law in absolute terms (mean difference of 0.67 points from a power score of 1–10), and relative to their mothers-in-law (mean difference in power score share of 5 pp), while the food intervention effects were weaker. Exploratory mediation analyses show that pregnant women’s absolute bargaining power, and their power relative to their mothers-in-law, can both mediate intervention effectiveness, but in slightly different ways.

Could this bargaining pathway be confounded by effects on the household budget? Households in the cash transfer arm did consume less staples and more (expensive, micronutrient-rich) animal-source foods overall, relative to the comparison group, while fruit and vegetable consumption was unchanged. However, we find no evidence that these effects mediate the effects of the cash transfer on intra-household allocation, and no association between these measures of the household food budget and bargaining power.

What else explains the effects of the cash intervention? The proportion of effect explained by changes in bargaining power is relatively small – at around 14%. This could be simply because we are decomposing a fairly small average effect and there is wide uncertainty in these mediation estimates, or because other mechanisms are also at play. The participatory women’s groups aimed to increase nutrition knowledge, but knowledge scores did not differ from the comparison group suggesting that this mechanism was not activated. However, group facilitators who provided the cash transfers deliberately ‘labelled’ the cash as belonging to the pregnant women. This may have enabled women to be given larger shares of foods purchased with the cash transfers without needing to bargain for it (Gram et al., 2019b). Taken together, we conclude that effects of the cash transfer on allocative behaviour can be (at least partly) explained by intra-household bargaining and perhaps also ‘labelling’ of the transfers.

How can we explain the effects of the food transfers on the allocation of staple foods? We find no evidence that the effects were mediated by changes in bargaining power, households’ total consumption, or nutrition knowledge. However, we show that staple food consumption declines with rising wealth, and the food transfer was particularly inferior. We posit that the staple food was channelled to these junior women because it was an inferior good, it was not preferred by other household members, and because it was also labelled as ‘pregnant women’s medicine’.

Our results have important implications. Firstly, the large in-equalities in intra-household food allocation indicate that interventions delivered at the household level may disproportionately benefit senior male members without careful programmatic design to change household preferences and/or bargaining power. Second, we show that this careful programming is possible; household allocative behaviour can be altered by well-designed interventions. However, the differences in ways that food and cash transfers affect food allocation illustrate how interventions can vary in their effects on women’s bargaining power, and in how ‘gender-transformative’ they are (Dworkin et al., 2015). In patriarchal contexts where young women have low levels of bargaining power, transfers of low-status inferior foods like fortified flour can increase nutritional equity without addressing patriarchal constraints that women face (not gender-transformative). On the other hand, transfers of cash can increase nutritional equity by altering the power dynamics between generations of women and increasing the bargaining power of junior women (gender-transformative). Third, interventions should consider the role of senior women in intervention development and evaluation. Interventions that increase younger women’s bargaining power may improve their health at the cost of older women rather than men. This may be acceptable to some extent: undernutrition in South Asia is far higher among younger women,^2^ and nutritional deficits during pregnancy have serious and intergenerational health consequences. However, adverse effects on older women in the household should be monitored.

The rest of the article is organised as follows. The second section describes the interventions and prior evidence for the hypothesised impact pathways. The third section describes the data collection, sampling procedures, and analytical methods. The fourth section describes respondents’ diets, estimates the effects of the food and cash interventions on food shares, and explores hypothesised impact pathways. The fifth section concludes.

## 2 The Low Birth Weight South Asia Trial

The Low Birth Weight South Asia Trial, LBWSAT, was a four-arm cluster-randomised controlled trial that aimed to improve birthweight and weight-for-age in children aged 0–16 months. The trial was registered with ISRCTN (ISRCTN 75964374) and full protocol published in Saville et al. (2016). This paper reports a secondary analysis of the trial, so we summarise relevant parts of the protocol in this section and provide any remaining reporting requirements of the CONSORT checklist in Appendix 1.

Eighty clusters (defined as Village Development Committees, VDC, administrative units) were randomly allocated to one of four trial arms:

(1) ‘PLA only’: Women’s groups using a Participatory Learning and Action (PLA) approach, facilitated by trained facilitators employed by a local NGO (Mother and Infant Research Activities, MIRA). There was around one PLA group per cluster per month. Facilitators guided participants through a cycle of meetings to identify and prioritise nutrition-related problems, learn together, identify solutions to these problems, and collectively act to address these problems.
(2) ‘PLA + cash’: Cash transfers of ~USD 7.5/month to pregnant women, delivered through PLA groups, in a system logistically supported by Save the Children Nepal.
(3) ‘PLA + food’: Food transfers of 10 kg/month of micronutrient-fortified wheat-soya-sugar blend, ‘Super Cereal’ (63.3% wheat flour, 25.0% soya bean flour, 10.0% sugar, 1.7% micronutrients), delivered through PLA groups in a system logistically supported by World Food Programme Nepal.
(4) ‘Control’: Standard government services.

Current evidence of effectiveness of these intervention components is mixed. Cash transfers and food transfers have shown some increases in child nutritional status but evidence on women’s diets and relative allocations within households is thin (Bastagli et al., 2016; Gentilini, 2014; Imdad and Bhutta, 2012; Manley et al., 2020; Ota et al., 2015). Food transfers are more cumbersome to administer than cash, so evidence showing that cash transfers can be similarly effective at alleviating undernutrition would provide support for a programmatic shift from food to cash in places with well-functioning markets. PLA groups have shown large reductions in maternal mortality in several low-income settings (Prost et al., 2013) and modest improvements in maternal diets but not nutritional status (Kadiyala et al., 2021; Nair et al., 2017).

The LBWSAT impact evaluation showed that PLA groups alone did not increase birthweight, diet diversity, or allocation of dietary energy to pregnant women (Harris-Fry et al., 2018; Saville et al., 2018). PLA + cash did not significantly affect birthweight but did improve women’s dietary diversity, whereas the PLA + food intervention improved birthweight and increased pregnant women’s allocation of energy but did not affect their diet diversity. Small effects on some dimensions of pregnant women’s agency were found in a sample with both joint and nuclear households (Gram et al., 2019a). Effects on intra-household shares of foods, and intergenerational power dynamics between mothers-in-law and daughter-in-law have not previously been reported.

In this study we report the impacts of the food and cash transfer interventions on pregnant daughters-in-law’s ‘food shares’ (daughters-in-law vs. mothers-in-law and daughters-in-law vs. male household heads), relative to a comparison group. We then explore whether effects on bargaining power may explain these effects, as well as possible alternative pathways by which these interventions may have affected food shares.

To identify which pathways to explore, we draw on the ‘collective model’ of household allocative behaviour wherein household members can have different preferences for how household resources should be allocated, and members’ relative bargaining power can influence these allocations (Bourguignon and Chiappori, 1992). The collective model yields a demand function for each food that is determined by bargaining power, household budget, preferences, and prices. At such low value, the cash and food transfers were unlikely to have affected prices. However, effects on bargaining power, budget, and preferences are possible. These three paths capture the main processes in the trial’s published Theory of Change (Saville et al., 2016). We describe these three hypothesised paths in turn.

### 2.1 Path 1: bargaining power

Studies have shown that the provision of cash transfers to women can increase indicators of women’s bargaining power (Almås et al., 2018; Ambler and De Brauw, 2017; Bonilla et al., 2017), and this in turn can explain increases in household food expenditures (Armand et al., 2016; Tommasi, 2019). Effects of cash transfers on the relative bargaining power between older and younger women, however, has not been well studied. Although there is some evidence that food transfers can also empower women (Buller et al., 2016), a comparative review of evidence suggests that cash transfers are more empowering for women (Gentilini, 2014).

In LBWSAT, the food and cash transfers were exclusively provided to pregnant women, to increase the likelihood of the transfers being controlled by and channelled to these women. The cash transfers were hypothesised to increase the relative bargaining power of daughters-in-law more than food transfers, because flour is considered inferior to rice and not safely saved for long periods, and Super Cereal is not widely available in markets and is less fungible than cash. This means that women would not have the same freedom to decide how to spend the Super Cereal as they would the cash.

We hypothesised that the provision of cash transfers would increase pregnant women’s bargaining power, and therefore increase their shares of food. The selective provision of cash to pregnant women could have increased their bargaining power in three ways. Firstly, giving women cash could increase their relative contribution to household income, which could in turn increase their decision-making power and control over allocative decisions. Second, women could save the nine transfers to provide a total one-off sum of NPR 6750 (USD 67.5) (Gram et al., 2019b). This money may have been particularly empowering for couples who were at the margin of affording separation from their in-laws. Giving cash to pregnant women in this position could have further strengthened their ‘outside options’, enabling them to bargain for better treatment and larger food shares. Third, it is possible that cash transfers changed the balance of power and young women’s control over allocative decisions through the signal that the cash sent. The act of an external organisation providing young women with cash, bypassing the usual gatekeepers of mothers-in-law or husbands, could send a normative signal that they should control cash in a context where this is quite unconventional (Gram et al., 2018). This extra-household support from group facilitators who provided the cash could have strengthened women’s bargaining power by placing social pressure on households to allow women to spend the cash according to her preferences.

The PLA groups (a component of both the food and cash transfer interventions) could have also increased women’s bargaining power by building friendships, extra-household support, and confidence. Others have shown that PLA groups can increase women’s decision-making power and self-confidence (Morrison et al., 2010).

### 2.2 Path 2: household food budget

A long literature has shown how cash transfers can drive a right-hand shift in the budget constraint, as measured by increases in household food consumption, expenditure, and security (Ahmed et al., 2019; Chakrabarti et al., 2020; Grijalva-Eternod et al., 2018; Raghunathan et al., 2017). Comparisons of food and cash transfers have shown that food transfers can also increase household food budgets and alter the composition of the food budget, but in different ways to cash transfers (Ahmed et al., 2019; Hidrobo et al., 2014; Hoddinott et al., 2018). These differences in impacts are not easily generalisable because of the wide variation in context, transfer size, and additional intervention components such as conditionalities, behaviour change communication, and ‘labelling’ of transfers (Gentilini, 2014).

In LBWSAT, the food and cash transfers were provided to shift the budget constraint, improve women’s diets and nutritional status in pregnancy and, in turn, improve the nutritional status of their infants. We can use Engle’s Law and Bennett’s Law to predict how the household might spend their transfers. Since the transfers were designed to be inframarginal to the staple food budget,^3^ we would expect poorer households to spend more of the cash transfer, or budget availed by substituting staple foods with Super Cereal, on necessities like staple foods. On the other hand, less-poor households should spend more of the transfer on income-elastic goods such as non-food items or nutrient-rich ‘luxury’ foods like fruits or animal-source foods (Behrman, 1988; Clements and Si, 2018; Cornelsen et al., 2015; Hoddinott et al., 2018). However, since many other studies have shown that food and cash transfers are not equivalent, it is also possible that staple food transfers simply added to the staple food budget, while the cash transfers were spent on nutrient-rich foods promoted by intervention facilitators (namely fruit, vegetables, and dairy).

Despite clear evidence that resources can be inequitably allocated within households, few studies have shown how food or cash transfers are distributed within households, or how they affect food allocation more broadly. However, some observational research has investigated the relationship between the size of the household food budget and intra-household allocation of energy and staple foods. In South Asia, women may act as a buffer to conditions of chronic food insecurity (Babu et al., 1993; Behrman and Deolalikar, 1990). This results in lower allocations of staple foods to women (Harris-Fry et al., 2017), especially the youngest daughters-in-law (Palriwala, 1993). We could therefore expect the allocation of staple foods to be less equitable across age and gender in the poorest households in food insecure contexts. This inequity may be reduced by the predicted rise in staple food consumption caused by the food and cash transfers in these households. If (younger) women absorb food shortages by reducing their intake of staple foods to preserve food for male and older household members, then an increase in the availability of staple foods should allow (younger) women to increase their own relative consumption of staple foods.

### 2.3 Path 3: knowledge and preferences

The third way by which the interventions could affect food allocation is through effects on preferences for food, or caring preferences. For example, mothers-in-law (or other community members) may gain new knowledge about the nutritional needs of pregnancy, causing households to place greater importance on the diets of pregnant daughters-in-law.

Educational interventions such as mass media campaigns that only aim to change food choices and caring preferences (but not budgets or bargaining power) have shown positive effects on nutrition outcomes and child feeding behaviours (Graziose et al., 2018). Effects of these educational interventions on preferences are therefore preceded by changes in nutrition knowledge so, although preferences are usually unobserved, effects on preferences may be proxied by more easily measurable indicators of nutrition knowledge.

As mentioned, the food and cash transfers were provided at PLA groups. In these groups, women learned together about nutrition problems and solutions, and collectively implemented strategies to address these problems in their communities. Examples of group strategies included community dramas to raise awareness of the importance of good nutrition in pregnancy, home visits to women who were not permitted to attend the groups, and additional group meetings with men and older women. All women (including daughters-in-law and mothers-in-law) were welcome to attend the PLA groups and learn about the nutritional requirements of pregnancy. In the cash arm the groups also discussed how to spend the cash transfers, and in the food arm they discussed recipes for using the flour and why pregnant women should eat it. Any of this may have increased the positive utility the mothers-in-law (or other household members) attached to their daughter-in law’s consumption, causing households to change their allocative behaviour.

## 3 Data and methods

### 3.1 Sampling and attrition

Our study is located in Dhanusha and Mahottari districts, in the rural floodplains of Nepal. In this region, maternal undernutrition is among the highest in the country, with over a quarter of women being underweight (<18.5 kg/m^2^) (DHS, 2011).^4^ Qualitative research has shown that junior women in this context have limited bargaining power, and that mothers-in-law typically control food-related decisions (Morrison et al., 2017).

Eighty clusters were randomly allocated to one of four trial arms, stratified by cluster size and accessibility. Between Dec 2013 and Feb 2015, the trial enrolled 63,308 women for monthly menstrual monitoring, and detected 25,092 pregnancies. All married women aged 10–49 years who had not had tubal ligation or whose husbands had not had a vasectomy were eligible for menstrual monitoring, and all women with a positive pregnancy test or who were visibly pregnant were eligible to become trial participants.

For this study, we use dietary intake data collected between May and Sep 2015 from a subsample of 800 multigenerational households with pregnant women enrolled in the trial. The sampling frame was restricted to women who were in their third trimester of pregnancy, and living in male-headed households with their in-laws, so all sampled households contained one pregnant woman, one mother-in-law, and one male household head. The target sample size was calculated as 200 per arm, to detect a two-sided difference in energy allocation ratios from 0.9 to 1.0 (assuming 0.27 SD and intra-cluster correlation of 0.03), with 80% power and a type I probability of 5%.

We interviewed 805/1074 (75%) eligible households, and include 800 in our analytical sample.^5^ In each household, we collected individual dietary recall of enrolled daughters-in-law, their mothers-in-law and male household heads, up to three times each, on non-consecutive days (6723 person-days; 2400 individuals; 800 households).

### 3.2 Measures of dietary intakes

Diets were measured using standard 24-h dietary recall protocols (Ferguson et al., 1995). Because diets have wide intra-individual variability and a 24-h recall provides a poor estimate of usual diets (Dodd et al., 2006), we measured intakes three times per person on non-consecutive days but within two weeks. Interviewers elicited respondents’ consumption using an atlas of graduated portion size photographs to aid estimation that we developed and validated locally (Harris-Fry et al., 2016), and the ‘multi-pass’ method involving multiple probes that has been shown to reduce under-reporting (Moshfegh et al., 2008). A food composition table was compiled from multiple national databases (Nepal, India, Bangladesh, US, and UK), and combined with locally collected recipe data to convert foods into nutrients.

We focus on the allocation of three key food groups: starchy staples (mainly rice, wheat, and potatoes), fruits and vegetables, and animal-source foods (dairy, meat, fish, eggs). We focus on staple foods because they constitute most of the diet and are crucial for achieving both macro- and micronutrient adequacy, whereas fruits and vegetables and animal-source foods were chosen because they are important sources of micronutrients but have different social meaning and economic value so could respond to changes in bargaining power or household availability in different ways.^6^ We check consistency of results by looking at dietary diversity (a count of 10 food groups per person (FAO, 2014)) that gives an overall measure of dietary variety and is an indicator of multiple micronutrient adequacy but does not capture differences in quantities.

Following the National Cancer Institute method to predict grams/ day of ‘usual intakes’ (Kipnis et al., 2009; Tooze et al., 2010),^7^ we use the triplicate recall and remove the within-person variance. More details are given in Appendix 2. We then calculate daughter-in-law’s food shares as a proportion of the sum of (i) all three members’ intakes (for descriptive purposes only), (ii) daughters-in-law and mother-in-law, and (iii) daughters-in-law and male household heads.

To characterise diets, we also report nutrient intakes (energy, iron, and vitamin A) and nutrient adequacy (accounting for differences in nutritional requirements) using data only from the control arm, and we describe usual allocative behaviour by showing kernel density estimates of shares of predicted usual intakes using an Epanechnikov kernel.

To estimate effects of the interventions on food shares, we do not account for differences in nutritional requirements because the requirements are calculated based on factors that the interventions will not affect (age, sex, pregnancy status).^8^

The National Cancer Institute method of predicting usual intakes relies on the assumption that observed recalls are unbiased estimates of true usual intake. In practice, recalls often underestimate. As one robustness check, we compare results with (*n* = 800) and without (*n* = 739) outliers (Tooze et al., 2012)^9^ Additionally, we use an anthropometric measure of nutritional status, mid-upper arm circumference (MUAC, cm), which is an objective measure of chronic energy deficiency that should corroborate results for staples.^10^

### 3.3 Measures of household food consumption, bargaining power, and knowledge

Household-level consumption for each food group (staples, fruits and vegetables, and animal-source foods) is indicated as the percentage share of total consumption of all foods. This is calculated as the grams of each food group consumed by all three measured household members as a percentage of the total grams of all foods (including staples, fruits, vegetables, animal-source foods, legumes, nuts, and seeds) consumed by all three household members.

We use two measures of bargaining power: one absolute and one relative. Absolute bargaining power is measured using a self-reported score from the ‘Power Ladder Question’ whereby daughters-in-law were asked to rate their perceived agency and control over life decisions between steps 1 and 10 on a ladder. This score is deliberately openly interpreted, allowing the respondent to decide what aspects of their lives contribute to their overall power (Lokshin and Ravallion, 2005). Since we are interested in investigating the importance of bargaining between daughters-in-law and mothers-in-law, we also calculate a relative measure of bargaining power. This is given as the daughter--in-law’s ‘power share’, which is her score as a proportion of the total for the two women. Perfect equality is 50%. We did not ask this question to male household heads, so we are unable to investigate the role of relative gendered power dynamics.

We use nutrition knowledge as a proxy for preferences. Nutrition knowledge was measured as a count of 20 items that measures respondents’ ability to list micronutrient-rich foods to eat in pregnancy and the health consequences of poor diets.

### 3.4 Estimating effects of food and cash transfers on intra-household food allocation

We estimate intent-to-treat effects of the food and cash transfers on daughter-in-law’s food shares relative to their mother-in-law and male household head by fitting multilevel linear regression models using maximum likelihood. We treat clusters as random effects. Shares of foods F between daughter-in-law (person A) and mother-in-law or household head (person B) is given as, $\frac{\zeta_{A}}{\zeta_{A}+\zeta_{B}}$, so the effect of the transfer interventions on food shares in household *i* from cluster *k* is defined as α_1_ in (1):

(1) $${\left\{\frac{\zeta_{A}}{\zeta_{A}+\zeta_{\text{B}}}\right\}}_{ik}^{\text{F}}=\alpha_{0}+\alpha_{1}t_{\text{i}k}+\alpha_{2}X_{\text{i}k}+u_{k}+\varepsilon_{ik}$$

We report cluster robust standard errors, which are clustered at the VDC level. *u_k_* denotes a random effect on the intercept, and *ɛ_ik_* is a cluster-specific random error for the household. We also control for a vector of socioeconomic covariates ***X***, identified as distinct determinants of food allocation in South Asia from a systematic review (Harris-Fry et al., 2017): caste group, wealth score,^11^ years of maternal education, a binary variable indicating whether the first interview was conducted before or during monsoon season (<Jul 17, 2015 or ≥ Jul 17, 2015 based on the date the rains came), and cluster randomisation stratum. Since clusters were allocated to treatments randomly, these covariates are included to increase the precision of the estimates, rather than to address risk of confounding; unadjusted results are also reported and are very similar, and variance inflation factors indicate any collinearity among predictors is not serious (all are <1.6).

We estimate the effects on hypothesised intermediary outcomes (bargaining power, household food consumption, and nutrition knowledge) in the same way, altering the dependent variable accordingly.

To describe heterogeneity in effects of the interventions on bargaining power and household budget, and we explore two possible effect modifiers: husband sending remittances from overseas (modifying effects on bargaining power) and wealth tertile (modifying effects on household budget). To do this, we extend the linear model given in (1) to include an interaction term between the intervention and hypothesised moderator.

### 3.5 Exploring impact pathways

We use a ‘potential outcomes framework’ to conduct mediation analyses that explore hypothesised impact pathways (Imai et al., 2010). To explain our approach, we use bargaining power as an example impact pathway. We let cash transfer be the exposure, bargaining power be the mediator, and food share be the outcome. We first estimate the food shares that would occur in the cash arm *with a bargaining power level that would occur in the cash arm*, and then subtract the counterfactual potential food share outcome that would occur in the cash arm *but with a bargaining power level as in the control*. In other words, we compare the difference in a household’s food shares for a fixed treatment status (being in the cash arm) but with different potential values of the bargaining power mediator. The difference between these two food share estimates gives us the indirect effect (termed ‘average causal mediated effect’ or ACME) of the treatment through the mediator. We implement this using the *‘mediation’* package in Stata, as in Hicks and Tingley (2011), which uses non-parametric simulations to estimate the counterfactual potential outcomes and their uncertainty.

These results are intended to be exploratory only. Inferring a causal mechanism through the mediator relies on ‘two assumptions of sequential ignorability’ (Imai et al., 2010). The first assumption is that the treatment allocation is independent of potential outcomes and mediators – this assumption is satisfied here since the allocation was randomised. The second assumption is that the mediator is ‘ignorable’ given the observed treatment status and covariates. In our case we have no way to confirm that this assumption is satisfied. For example, our analyses explore each pathway separately, but they could be interrelated and confound each other: increases in the household budget could increase both women’s bargaining power and food shares, or increases in bargaining power could cause households to alter their food budget and food allocation. We perform sensitivity analyses to examine how the estimated indirect effect will change according to different levels of correlation between the error terms in the two models (mediation and outcome models), and how large this correlation needs to be for the indirect effect to disappear.

All analyses were conducted in Stata SE 17 (StataCorp LP) apart from the prediction of usual intakes, which was implemented in SAS University Edition using the National Cancer Institute’s macros (MIXTRAN and INDIVINT).

## 4 Effects of food and cash transfers on intra-household allocation

### 4.1 Respondent characteristics, diets, and intra-household allocation

Household and individual-level characteristics of the sample are summarised by treatment in Table 1, and pooled estimates are described in text. Consistent with the high levels of poverty and poor educational facilities in rural Nepal, education levels are low. Around a third of households are landless (28%) and from socially disadvantaged groups (Muslim and Dalit caste groups) (30%). Overseas migration is common, with around 20% of households having at least one member living overseas. Intra-household differentials are observed in terms of age and education. As expected, daughters-in-law are younger than their mothers-in-law and male household heads, by around 30 and 20 years, respectively. Wives are also less educated than their husbands. Over half the wives surveyed (54%) have no education, compared with 37% of husbands.

**Fig. 1:**
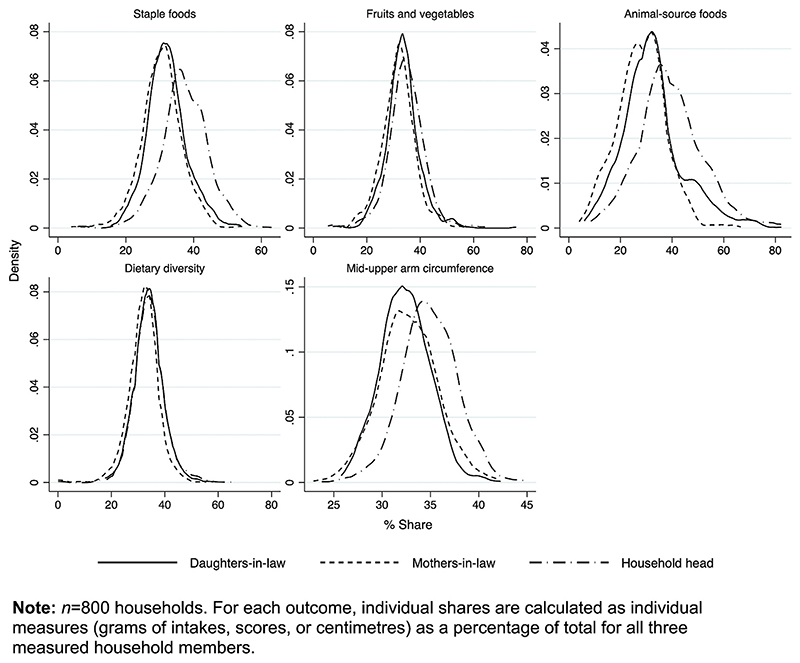
Kernel density estimates of shares of nutrients, foods, diversity, and nutritional status allocated to different household members. **Note:** *n =* 800 households. For each outcome, individual shares are calculated as individual measures (grams of intakes, scores, or centimetres) as a percentage of total for all three measured household members.

Table 2 describes the dietary behaviours and nutritional outcomes of each household member in the control arm, and Fig. 1 illustrates within-household allocation, showing kernel density estimates of shares of foods and nutritional status by household member.

There are notable differences in food-related behaviours by gender and generation. Compared with women, male household heads are more likely to go out to buy food (40%), but less likely to make decisions about (22%) or prepare food (0%). Between generations of women, more daughters-in-law are the primary cook (77% vs 3%), but fewer are involved in decisions about food (32% vs 61%).

We find gender disparities in the allocation of staples, animal-source foods, and nutritional status, while the diet diversity and quantities of fruit and vegetables are more evenly distributed. Allocations between generations of women are similar. Given the nutritional demands of pregnancy, this allocation creates a gradient within the household, wherein dietary adequacy of male household heads > mothers-in-law > daughters-in-law. For example, average energy requirements were not met in 38% of daughters-in-law, 18% of mothers-in-law, and 17% of male household heads. When we account for self-reported physical activity, this inadequacy rises (daughters-in-law 53%; mothers-in-law 36%; household heads 42%). Reflecting this inequity, a larger proportion of women (mothers-in-law: 35%; daughters-in-law: 40%) than men (14%) are classified as thin (MUAC <23 cm (Tang et al., 2013)).^12^ Additionally, all daughters-in-law, many mothers-in-law (64%) and significant number of household heads (23%) have very low (<1%) probability of consuming adequate dietary iron. This indicates that households (over) account for the energy requirements of being male and physical activity levels, but not the iron needs from menstruation or energy or iron requirements of childbearing.^13^

Vitamin A intakes appear adequate, probably because the sampling period (May to Sep) includes mango season. Strong seasonal effects have been reported in Nepal, showing a sharp peak in consumption of vitamin-A rich fruits (Saville et al., 2021) and serum beta-carotene concentration (Jiang et al., 2005) over this season.

To our knowledge, LBWSAT is the only study to have measured diets of mothers-in-law and daughters-in-law, giving new insight into behaviour of joint households. However, the gender differentials echo findings from other South Asian studies (D’Souza and Tandon, 2019; Gittelsohn et al., 1997; Sudo et al., 2006).

### 4.2 Effects of food and cash transfers on food shares

Given the inequity in intra-household allocation described in this context, interventions could potentially improve the health outcomes of young pregnant women by affecting household allocative behaviour. As we described in Section 2, the cash and food transfers tested in LBWSAT aimed to do this; here we examine whether they did.

Respondent characteristics across arms indicates the trial arms are generally well balanced (Table 1) with non-differential attrition (Appendix Table A1).

In the control arm, 1.6% of households attended any PLA meetings, indicating minimal contamination. Intervention coverage was high in both food and cash transfer arms, with most women receiving four or more transfers (PLA + cash: 98%; PLA + food: 93%). In contrast, only 4% of women attended four or more PLA meetings in the PLA only arm. Given this much lower attendance, and because we are particularly interested in the effects of the transfers on power dynamics and food consumption, we focus on the effects of the PLA + cash and PLA + food arms and pool the control with the PLA only arm to give a comparison group with more statistical power. Comparisons using the control arm only show similar results with wider confidence intervals.

Intent-to-treat estimates of the effects of the PLA + cash and PLA + food interventions on food shares, each relative to the comparison group, are given in Table 3. Very similar unadjusted results are reported in Appendix Table A2.

We show that, relative to the comparison group, households in the PLA + cash arm allocated daughters-in-law with 2 pp [95% CI 0.6 to 3.6] larger shares of staples and 2 pp [0.2 to 3.2] larger shares of fruit and vegetables relative to their mothers-in-law, and 3 pp [0.6 to 6.1] larger shares of animal-source foods relative to male household heads. This is equivalent to an increase of 0.26, 0.20, and 0.22 standard deviations in shares of staples, fruits and vegetables, and animal-source foods respectively. Results are corroborated by daughters-in-law having larger gains in MUAC (an indicator of energy adequacy) relative to mothers-in-law but not relative to household heads (Appendix Table A3). These differences in gendered and intergenerational effects suggest that the allocations of different food types are differentially amenable to change, perhaps depending on whether the sociocultural status of the foods is lower (e.g. fruits and vegetables) or higher (e.g. animal-source foods).

In contrast, the food transfer intervention only increased daughter-in-law’s allocation of staples relative to mothers-in-law (by 2 pp [95% CI 1.1 to 3.4]), which corresponds to an increase of 0.28 SD in shares of staples. The allocation of other foods did not change. These effects are not corroborated by similar effects on MUAC (Appendix Table A3), but they do mirror intra-household differences in the percentages of individuals consuming any of the Super Cereal in the PLA + food arm (pregnant women 54%; mothers-in-law 12%; male household heads 6%).

This suggests that, while both interventions arms received transfers of a similar value and ran similar PLA groups with similar levels of population coverage, these interventions worked differently.

In Table 4 we report the effects of the food and cash transfer interventions on intermediary outcomes that we hypothesised to be on the impact pathway, causing larger shares of food to be allocated to daughters-in-law. These are bargaining power of daughters-in-law, household food budget, and nutrition knowledge.

The results show that the cash transfers increased the absolute and relative bargaining power of daughters-in-law, whereas much weaker effects are observed in the food arm, as described below. The cash transfers also altered the household food budget, while the food transfers did not. Nutrition knowledge did not improve in either treatment. Could these different effects on bargaining power and/or household food budget explain the differential effects on intra-household food allocation? We examine each pathway in turn.

### 4.3 Bargaining power

The results in Table 4 show that the cash transfers affected power balances within the household, resulting in daughters-in-law having around 0.7 [95% 0.2 to 1.2] steps higher on the self-reported power score, and 5 pp [1.1 to 8.6] higher shares of bargaining power relative to their mothers-in-law. In the food arm, daughters-in-law had slightly higher power scores (0.4 steps [–0.01 to 0.9]), but power shares did not differ. This corroborates our hypothesis that the cash transfers would affect power balances more than food transfers, and that cash might not just increase the bargaining power of daughters-in-law but could also reduce the power of mothers-in-law as they lose (some of) their traditional role in controlling food expenditures and caring for their daughter-in-law.

If this relatively small amount of cash is empowering, we could expect to see smaller effects in households with higher incomes. We explore this in Appendix Table A4 by looking at differential impacts on bargaining power, depending on whether the spouse worked overseas. In this context, overseas remittances are a major source of household income, and can drive wide heterogeneity in household wealth. As expected, we find significantly smaller effects of the PLA + cash on power shares when the spouse lives overseas (–2 pp) than when they do not (+6 pp) (test for interaction *p =* 0.040), although confidence intervals are wide. This differential effect is in line with qualitative research that indicates that the cash transfers were less empowering in households that were already relatively well-off because they were receiving remittances (Gram et al., 2019b).

Do these effects on bargaining power explain the effects on intra-household food allocation? The results from Table 5 suggest they mediate effects of cash transfers but not food transfers – and this mediation of cash effects varies depending on whether we look at absolute (daughter-in-law) or relative (intergenerational) bargaining power.

The absolute measure of daughter-in-law’s bargaining power partially mediates cash effects on the allocations of fruits and vegetables between women (indirect effect [95% CI]: 0.24 [0.02, 0.64]) and the allocation of animal-source foods between women and men (0.42 [0.02, 1.26]). Intergenerational bargaining power also explains some effect on intergenerational fruit and vegetable allocation (0.24 [0.01, 0.56]) – to a similar extent as absolute bargaining power. However, it also explains the effects on allocations of staples between generations of women (0.28 [0.01, 0.63]) (which the absolute measure did not find) while showing no role in mediating the gendered allocations of animal-source foods.

We interpret this as evidence that cash transfers can affect inter-generational bargaining within households, and that intergenerational bargaining power can mediate the effects of cash transfers on household allocative behaviour in slightly different ways to absolute measures of bargaining power. We interpret our mediation results tentatively, given the risk of confounding between mediator and outcome described in Section 3.5. Sensitivity analyses (Appendix Table A5) show the correlation between error terms of the mediator and outcome would need to be around 0.1 for the indirect effect to disappear. The most obvious concern is that the effects on food budgets are confounding this indirect effect, although later analyses in section 4.4 suggest that this is not the case.

It is also important to note that bargaining power only explains about 14% of the effects on the allocation of foods (for all foods studied). This may be due to wide variance and measurement error for these mediators and outcomes and because we are decomposing a relatively small effect, or it may be that other pathways through food budget or preferences are also responsible.

### 4.4 Household food budget

Do effects on food budgets also explain these effects on food allocation? Our results show that households in the cash transfer arm substituted cheaper, more energy-dense staples with more expensive and micronutrient-rich animal-source foods. The household food basket in the cash arm contained 5 pp lower shares of staple foods but 4 pp larger shares of animal-source foods, while shares of fruits and vegetables remained similar to the comparison group.^14^ This increased consumption of animal-source foods was expected, and corroborated by qualitative research from the trial (Gram et al., 2019b). Animal-source foods are an important source of multiple micronutrients required in pregnancy. In particular, milk is sold by door-to-door sellers, thereby overcoming barriers women face in leaving their homes in this context. In contrast, fruit and vegetables usually need to be purchased at markets, so would rely on support from other household members. Additionally, fruits are more expensive than milk; one month’s cash transfer would buy 30 L of milk but only 4–7 kg of apples or 3-4 kg of pomegranates (Gram et al., 2019b). Given the high levels of chronic energy deficiency in the region, the lower consumption of staple foods was an unintended consequence of the cash transfer intervention – it was hoped that the cash transfers would increase total consumption rather than cause households to substitute foods.

In contrast, the food transfer intervention did not affect household shares of staples, fruits and vegetables, or animal-source foods. This is surprising because we expected that the food transfers would supplement the diets, perhaps leading to higher staple food consumption, or at least availing resources to buy more non-staple foods.

**Fig. 2:**
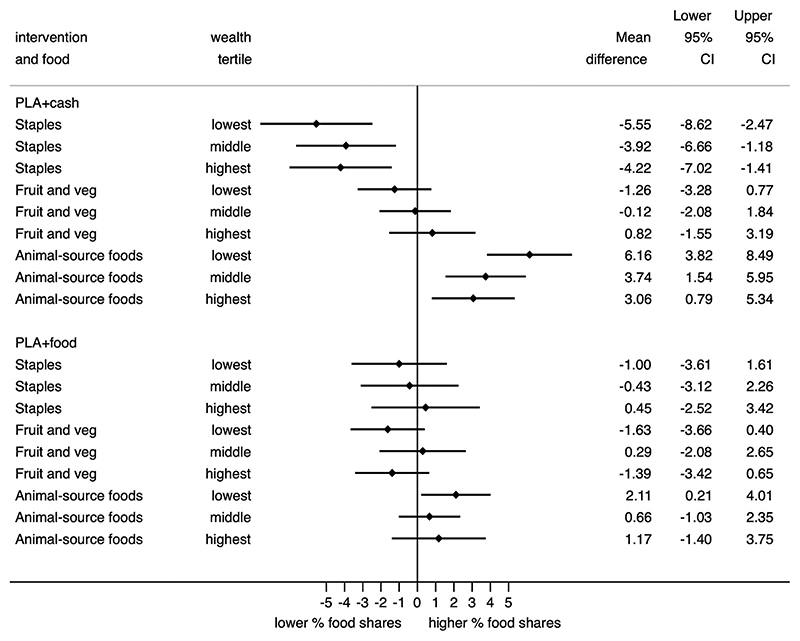
Forest plot of the effect of food and cash transfers on household food shares stratified by wealth tertile.

The null average treatment effect of the food and cash transfer interventions on household-level consumption of staple foods could be explained by heterogeneity in effects by household wealth. We hypothesised in Section 2 that, if staples were inferior goods, the transfers would increase the consumption of staple foods in poorer households. Equivalently, if fruits, vegetables, and animal-source foods were normal (or comparatively ‘luxury’) goods, the transfers would increase consumption of these foods in better-off households. Analyses of control arm data confirm that household shares of staple foods decline with rising wealth, whereas shares of fruit and vegetables and animal source foods rise with increasing wealth.^15^ However, sub-group analyses show no consistent differences between wealth tertiles in the effects of cash or food transfers on household food consumption (food shares) (Fig. 2).

There are a few possible explanations for the limited effect of the Super Cereal on the food budget. One possible explanation is that there was low compliance to the intervention due to very low preferences for the transfer, and that the pregnant women had become tired of consuming it every day by the time they reached their third trimester. In short, it is possible that the Super Cereal was not ‘liked’. Although around half of the pregnant women (54%) in the food transfer arm consumed at least some Super Cereal on measurement days, only 3% consumed the recommended 150 g/d. It is also possible that the food transfers increased consumption of staples (including Super Cereal) in other, unmeasured household members, such as children.

The lack of effect of the PLA + food intervention on food budget indicates that this food budget pathway does not explain effects on intra-household food allocation. However, the PLA + cash intervention effects on household budget could explain the effects on intra-household food shares. We explore this in Table 6. The results show very low, non-significant indirect effects of cash transfers on food allocation through the household-level consumption indicators.

Furthermore, additional analyses show no evidence of an association between intergenerational bargaining power and household consumption of staple foods (–0.16 [95% CI -0.43 to 0.11], *p =* 0.243) or animal source foods (0.06 [–0.21 to 0.33], *p =* 0.651), indicating that the effects on food budget are not confounding the bargaining power pathway. The same is true with absolute levels of bargaining power.^16^

### 4.5 Knowledge and preferences

Finally, we examine whether the intervention affected nutrition knowledge, measured as a score of 20 items. We find no effect in either transfer arm (Table 4), so do not explore mediation any further.

It is possible that our measure of nutrition knowledge was not sensitive enough. It is well-known that nutrition knowledge is difficult to measure well (Nutbeam, 2009), so measurement error could explain these null effects. However, the lack of effect may also be because the PLA component, the key conduit for knowledge development, ended up being only weakly implemented, especially at the time these diet data were collected (at the end of the trial when enthusiasm of staff and participants may have waned). Qualitative process evaluation also reported that the group functioning in the transfer arms was compromised by the distraction of administering the transfers (Morrison et al., 2020). The trial was also implemented during the 2015 earthquakes and severe political conflict during the Federalization process.^17^

Other unmeasured effects on preferences may also play a role. In particular, the transfers were ‘labelled’ as belonging to the pregnant women. This means that household members may have had different preference functions for the transferred food (or food purchased with cash transfers) compared with other household food. This may resolve the so-far unexplained effects of food transfers on the allocation of staple foods, and the remaining effect of cash transfers on food allocation.

For food transfers, we speculate that the effect on intra-household staple allocation was driven by low preferences for the Super Cereal (in general) but comparatively higher preferences for it among pregnant women. In the PLA meetings, facilitators deliberately branded the Super Cereal as being a ‘pregnant woman’s medicine’ that could be easily channelled to junior women with low bargaining power without challenging existing household hierarchies. This may have caused households to allocate daughters-in-law relatively more staples, and perhaps compensate other members with larger shares of other unmeasured goods.

Facilitators who administered the cash and ran the PLA meetings also branded the cash as ‘belonging to the pregnant woman’. Therefore, the cash might have been spent on animal-source foods for pregnant women without need for any negotiation or additional bargaining power. Labelling is a common addition to cash transfer programming, sometimes called a ‘soft condition’, that has explained cash transfer effects in other places (Bastagli et al., 2016). Analyses of data on participants from the cash arm show that most daughters-in-law controlled their cash transfers, with 67% of women reporting that they were involved in decisions about how the cash should be spent, which is much more than the usual involvement in spending decisions (13%). This is consistent with the qualitative research from the trial which reports that the pregnant women spent the cash on animal-source foods (particularly on milk and curd) that they ate for themselves (Gram et al., 2019b). This was not only because they were more empowered, but also because they were more likely to make decisions about how this specific cash should be spent, and because it was earmarked for their use by the program implementers.

## 5 Conclusion

We unpack household allocative behaviour in a resource-constrained setting of rural Nepal. Using dietary data on pregnant daughters-in-law, mothers-in-law, and male household heads, we identify intra-household food allocation rules and the role of intergenerational bargaining power in determining the effects of food and cash transfer interventions on these allocation rules.

We show that diets are generally highly inadequate and inequitably allocated within households in this setting. Dietary intakes do not meet the nutritional requirements of macro- and micronutrients necessary for good health. Iron and energy deficiencies are concerning, with most women and men having very low dietary iron adequacy. Consistent with other literature (D’Souza and Tandon, 2019; Gittelsohn et al., 1997; Sudo et al., 2006), we show that men receive the lion’s share of the food budget, even after accounting for differential requirements due to physical activity. We also reveal previously unknown similarities in the relative allocation of food between mothers-in-law and daughters-in-law. Households do not appear to compensate for the elevated requirements of pregnancy, resulting in higher micro- and macronutrient dietary deficiencies in pregnant daughters-in-law than other household members. This implies that, without careful design, interventions delivered at the household level may by default disproportionately benefit men.

We also show that nutrition interventions can be designed to influence these allocative behaviours, and help to reduce intra-household inequities in dietary adequacy. However, the ways that interventions achieve this can vary. The provision of inferior but micronutrient-rich Super Cereal can be channelled to lower status, junior women, perhaps with the help of behaviour change communication and transfer labelling or branding. This can reduce gender gaps in dietary inadequacy, but does so without challenging the patriarchal status quo, meaning that these interventions are effective at improving nutritional outcomes (Saville et al., 2018) in spite of (or perhaps because of) the low relative bargaining power of junior women. Food transfer programs providing different food baskets, such as rice, might be less easily channelled to lower status women within the household, as has been shown in a comparison of wheat versus rice transfers in food-for-work schemes in Bangladesh (Ahmed et al., 2007). Cash transfers, on the other hand, may be classified as a ‘gender-transformative’ intervention because they can increase the equity in the allocation of multiple foods (in part) by increasing the relative bargaining power of junior women within the household (Dworkin et al., 2015). Although we cannot make causal claims about these bargaining processes, our exploratory analyses indicate that effects on intergenerational bargaining power can mediate the effects of the cash transfers. This indicates that analyses of joint households should not be reduced to two-person, husband-wife frameworks, and that the role of mothers-in-law should be factored into the design of interventions aiming to reach and/or benefit junior women living in joint households.

Anthropological literature has documented that many South Asian women internalise the prevailing cultural norms of pro-male bias, gaining satisfaction from nourishing their family, and choosing to be self-sacrificial to signal honour and respect to their family (Messer, 1997). Whilst this indicates that these women may have weaker preferences for their own wellbeing – an issue that Amartya Sen and many feminist scholars have articulated (Sen, 1987) – our results suggest that women will allocate themselves more food when they can.

There are some important differences in the ways that household allocative behaviour changes in response to cash transfer interventions. In particular, the cash transfers affected allocations of fruits and vegetables between generations of women, but they affected the gendered allocations of animal-source foods. This suggests that there are differences in the negotiability of food allocation in this context. Given that our descriptive results show women (both mothers-in-law and daughters-in-law) are involved in food-related processes in the household, food allocation between women might be more amenable to change. In contrast, in this context men do not tend to spend time in the kitchen and are typically served and eat first until they are satisfied, so they will not see how little is left or observe allocation decisions (Morrison et al., 2021). This may explain why gendered allocation of animal-source foods were affected by bargaining power but other foods were not: being only occasionally consumed, the quantity of the animal-source foods available may be more publicly known. Or, men may be more inclined to find out how much there is and ensure there is enough left for the daughter-in-law when she has more bargaining power.

The study strengths and limitations warrant further discussion. This study uses a unique dataset that provides new insight into intergenerational differentials in bargaining power and food allocation in joint households. We measured this in the context of a randomised trial, which enabled us to identify whether and how these factors are amenable to change. However, our exploratory analyses of the role of bargaining power in mediating intervention effects should be considered with the caveat that we did not measure diets or bargaining power at enrolment and cannot rule out confounding of the mediator-outcome relationship. Additionally, we did not measure bargaining power of men in the household so we are unable to directly compare the differences in relative gendered and intergenerational bargaining power.

Our findings can be used to inform how poverty alleviation and public health programs delivered at the household level can both empower and benefit junior women, and the conditions under which men and senior women may reallocate their larger shares of household resources. Previous studies have shown that interventions aiming to increase women’s bargaining power do not always benefit women, highlighting the need to monitor effects on intended and unintended outcomes. For example, asset transfer programs can increase women’s workloads (Johnson et al., 2016); income generation can be a risk factor for violence against women (Vyas and Watts, 2009); and equal land inheritance laws can result in more son preference (Bhalotra et al., 2018; Rosenblum, 2015) and heavier workloads (Rao, 2006). Our findings highlight that these programs should not only monitor intended and unintended effects on young women and their spouses, but should also include older women within joint households in intervention design and evaluation.

## Supplementary Material

Appendix

## Figures and Tables

**Table 1:** Household and individual characteristics by arm.

	Statistic	*n*	Control	PLA	PLA + cash	PLA + food
	*n*	800	148	153	281	218
Muslim or Dalit (disadvantaged)	Proportion	800	0.35	0.32	0.29	0.27
Household owns land	Proportion	800	0.66	0.65	0.78	0.73
Member living overseas	Proportion	702	0.46	0.38	0.48	0.50
Household wealth score	Mean	800	−0.10	−0.16	0.20	−0.08
Household size	Mean	800	7.3	7.5	7.9	7.9
Monsoon season	Proportion	800	0.57	0.50	0.58	0.58
Age, daughter-inlaw	Mean	800	20.6	20.2	20.5	20.8
Age, mother-in-law	Mean	769	50.5	48.9	50.9	50.0
Age, household head	Mean	785	40.5	41.5	43.6	45.0
Education, years, husband	Mean	796	4.8	5.2	4.6	5.6
Education, years, wife	Mean	800	3.2	3.3	3.5	3.7
Wife more educated	Proportion	796	0.16	0.13	0.22	0.19
Spouse is head of household	Proportion	800	0.36	0.31	0.33	0.29

**Note:** Monsoon season defined as pre-monsoon (<Jul 17, 2015), or monsoon (≥ Jul 17, 2015), based on the date the rains came that year. Household wealth score = First principal component from 14 assets owned by household. Some variables are missing values because they were missed from the main surveillance system, or because respondents did not know their age.

**Table 2:** Dietary intakes, adequacy, and nutritional status by household member.

	Daughters-in-law		Mothers-in-law		Household heads	
	Centiles		Centiles		Centiles	
	50	[25, 75]	50	[25, 75]	50	[25, 75]
*Food intakes*						
Staples, g/d	859	[675, 1062]	799	[623, 1007]	1056	[818, 1329]
Fruit & veg, g/d	300	[217, 412]	326	[233, 447]	351	[249, 486]
Animal-source, g/ d	164	[80, 267]	132	[58, 226]	239	[140, 371]
Diversity score	5	[4, 5]	5	[4, 5]	5	[4, 6]
*Physical activity levels*						
Sedentary, %	8		6		4	
Moderate, %	91		68		56	
Strenuous, %	1		26		40	
*Nutrient adequacy*						
Energy, intake/ EAR ^a^	1.06	[0.91, 1.28]	1.31	[1.11, 1.59]	1.35	[1.13, 1.56]
Iron, Pr (adequate) ^b^	0.00	[0.00, 0.00]	0	[0, 0.06]	0.15	[0.04, 0.35]
Vit A, Pr (adequate) ^c^	0.76	[0.26, 0.99]	1.00	[0.84, 1.00]	0.99	[0.84, 1.00]
*Nutritional status*						
MUAC, cm	23.5	[22.1, 24.6]	24.0	[21.8, 26.6]	25.9	[24.0, 27.5]
Low MUAC, % <23 cm	0.40		0.35		0.14	
*Food-related activities*						
Is the primary cook, %	78		3		0	
Makes food decisions, %	33		55		22	
Goes outside to shop, %	13		35		40	

**Note:** *n* = 148 for each household member category (control arm only); MUAC = mid-upper arm circumference; Diversity score as defined by FAO & FANTA (2016).

a EAR = Estimated Average Requirements, calculated using the Schofield equation (FAO/WHO/UNU, 1985), assuming a Physical Activity Level of 1.6 for all household members (Srinivasan et al., 2020) and an additional cost of pregnancy of 390 kcal/d (ICMR, 2010).

b Estimated using a table of probabilities of adequacy for different intervals of usual intakes, assuming 5% bioavailability, or 15% if pregnant (Food and Nutrition Board & Institute of Medicine, 2001).

c Estimated by relating usual intakes to their population distribution of requirements, which are Normal distributions with mean (i.e., EAR) and standard deviation (FAO/ WHO, 2001).

**Table 3:** Intent-to-treat estimates of the effect of food and cash transfer interventions on food shares.

	Control & PLA	PLA + cash	PLA + food	PLA + cash vs. Control & PLA		PLA + food vs. Control & PLA	
	Mean (SD)	Mean (SD)	Mean (SD)	Adjusted mean difference [95% CI]	*p*-value	Adjusted mean difference [95% CI]	*p*-value
**Shares between daughters-in-law and mothers-in-law**							
Staples	50.1 (7.98)	52.1 (7.9)	52.1 (7.86)	2.06 [0.58, 3.55]	0.006	2.24 [1.06, 3.43]	<0.001
Fruit & veg	50.8 (8.40)	52.5 (8.31)	50.9 (8.45)	1.69 [0.19, 3.19]	0.027	0.26 [−1.48, 1.99]	0.771
Animal-source foods	52.3 (13.18)	54.1 (13.23)	53.6 (14.4)	1.70 [−0.37, 3.78]	0.108	1.38 [−1.14, 3.91]	0.282
**Shares between daughters-in-law and male household heads**							
Staples	46.0 (7.84)	46.2 (8.34)	47.3 (8.60)	0.15 [−1.15, 1.44]	0.825	1.41 [−0.18, 3.00]	0.081
Fruit & veg	48.9 (8.31)	49.5 (8.19)	49.0 (8.52)	0.64 [−0.72, 2.00]	0.358	0.16 [−1.38, 1.70]	0.837
Animal-source foods	43.7 (15.21)	46.7 (13.79)	45.9 (15.83)	3.34 [0.63, 6.06]	0.016	1.89 [−1.05, 4.83]	0.208
*n*				582		519	

Note: 95% CIs based on cluster-robust SEs. Controls: caste group, wealth score, education level of daughter-in-law, season, and cluster stratum.

**Table 4:** Intent-to-treat estimates of the effects of food and cash transfer interventions on intermediate outcomes.

	Control & PLA	PLA + cash	PLA + food	PLA + cash vs. Control & PLA		PLA + food vs. Control & PLA	
	Mean (SD)	Mean (SD)	Mean (SD)	Adjusted mean difference [95% CI]	*p*-value	Adjusted mean difference [95% CI]	*p*-value
**Bargaining power**							
*n*	301	281	218				
Absolute power, score of DIL from 1 to 10	4.2 (2.31)	4.8 (2.36)	4.6 (2.44)	0.67 [0.18, 1.15]	0.006	0.42 [−0.01, 0.86]	0.058
Relative power, DIL/(DIL + MIL) %	41.3 (17.33)	45.4 (16.08)	41.5 (16.97)	4.81 [1.05, 8.57]	0.012	0.59 [−2.37, 3.55]	0.696
**Household food budget (shares, as a % of all foods)**							
*n*	301	281	281				
Staples	57.5 (8.63)	52.1 (8.70)	56.6 (8.44)	−4.50 [−6.42, − 2.58]	<0.001	−0.22 [−2.15, 1.72]	0.826
Fruit & veg	23.4 (6.51)	23.9 (7.19)	22.6 (6.56)	0.22 [−1.06, 1.50]	0.732	−0.56 [−1.72, 0.59]	0.340
Animal-source foods	12.3 (6.56)	16.7 (7.36)	13.8 (7.48)	3.89 [2.31, 5.47]	<0.001	0.88 [−0.46, 2.22]	0.198
**Nutrition knowledge**							
*n*	265	256	183				
Knowledge score from 1 to 20	4.9 (2.79)	5.7 (2.69)	5.7 (3.54)	0.62 [−0.52, 1.77]	0.286	0.60 [−0.82, 2.03]	0.406

Note: DIL = Daughter-in-law; MIL = Mother-in-law. 95% CIs based on cluster-robust SEs. Controls: caste group, wealth score, education level of daughter-in-law, and cluster stratum. Nutrition knowledge was measured on the third dietary recall so there are some missing values due to loss-to-follow-up.

**Table 5:** Mediation of effect of food and cash transfers by bargaining power.

Treatment	Mediator	Outcome	Direct effect [95% CI] of treatment	ACME [95% CI]: Indirect effect through mediator
PLA + Cash	Absolute bargaining power (DIL power score)	Staple shares to DIL vs.MIL	1.90 [0.48, 3.38]	0.14 [−0.06, 0.55]
		F&V shares to DIL vs. MIL	1.47 [0.05, 2.96]	0.24 [0.02, 0.64]
		ASF shares to DIL vs. HHH	2.56 [−0.06, 5.29]	0.42 [0.02, 1.26]
PLA + Cash	Relative bargaining power (DIL vs MIL, % power share)	Staple shares to DIL vs. MIL	1.79 [0.30, 3.32]	0.28 [0.01, 0.63]
		F&V shares to DIL vs. MIL	1.47 [−0.04, 3.01]	0.24 [0.01, 0.56]
		ASF shares to DIL vs. HHH	2.64 [−0.11, 5.45]	0.28 [−0.11, 0.83]
PLA + Food	Absolute bargaining power (DIL power score)	Staple shares to DIL vs. MIL	2.12 [0.99, 3.31]	0.02 [−0.12, 0.29]

Notes: We only explore mediation if intent-to-treat effects are observed on both mediator and outcome. Abbreviations used: ACME: Average causal mediated effect; ASF: Animal source foods; CI: Confidence interval; DIL: Daughter-in-law; F&V: Fruit and vegetables; HH: Household; HHH: Household head; MIL: Mother-in-law.

**Table 6:** Mediation of effect of food and cash transfers by household consumption.

Treatment	Mediator	Outcome	Direct effect [95% CI] of treatment	ACME [95% CI]: Indirect effect through mediator
PLA + Cash	HH % share of staples	Staple shares to DIL vs MIL	1.72 [0.35, 3.13]	0.34 [−0.09, 0.89]
	HH % share of F&V	F&V shares to DIL vs MIL	1.63 [0.14, 3.15]	0.07 [−0.12, 0.34]
	HH % share of ASF	ASF shares to DIL vs HHH	3.04 [0.34, 5.81]	−0.12 [−0.55, 0.24]

Notes: We only explore mediation if intent-to-treat effects are observed on both mediator and outcome. Abbreviations used: ACME: Average causal mediated effect; ASF: Animal source foods; CI: Confidence interval; DIL: Daughter-in-law; F&V: Fruit and vegetables; HH: Household; HHH: Household head; MIL: Motherin-law.

## Data Availability

Data will be made available on request.

## Footnotes

1 Abbreviations used: LBWSAT = Low Birth Weight South Asia Trial; MUAC = mid-upper arm circumference; NPR=Nepalese rupees; PLA=Participatory Learning and Action; pp = percentage points; VDC=Village Development Committee.

2 For example, in India and Nepal, 42% and 30% of girls aged 15–19 years are underweight (body-mass index <18.5 kg/m^2^) respectively, whereas only 14% and 13% of women aged 40–49 years are underweight (India DHS, 2015–16; Nepal DHS 2016).

3 The food transfer provides 680 kcal/d, and the cash transfer was the equivalent value of the food. 680 kcal/d provides 29% of the energy requirements of the average pregnant woman in this context, assuming the average woman is 50 kg, aged 19–30 years, requires an additional 390 kcal to meet the energetic costs of pregnancy, and has a Physical Activity Level factor of 1.6.

4 According to 2011 Demographic and Health survey, 26% of women had low BMI in the Central Terai region, where Dhanusha and Mahottari districts are located. The more recent Demographic and Health Survey from 2016 used different zones due to the federalization of the country, and a different sampling strategy, so estimates are not comparable. But, in Province 2, 29% of women had low BMI.

5 Reasons for attrition were migration (*n* = 13), respondents not available (*n* = 219), unable to locate home (*n* = 1), declined to consent (*n* = 23), and no reason reported (*n* = 13). Of 805 interviewed households, we exclude 5 due to missing demographic data to predict usual consumption.

6 Staples are known to have lower food price elasticity than other more micronutrient-rich foods like fruits and vegetables or animal-source foods so could plausibly show different results to these other foods (see, e.g. Cornelsen L, Green R, Turner R, Dangour AD, Shankar B, Mazzocchi M, Smith RD. What happens to patterns of food consumption when food prices change? Evidence from a systematic review and meta-analysis of food price elasticities globally. Health economics 2015; 24; 1548–1559.) Animal-source foods are particularly considered to be high-status, special foods, compared with fruits and vegetables, so mechanisms by which household allocation of these food types could change may also be quite different.

7 The exception is energy; requirements are based on physical activity levels and weight, and these could be affected by LBWSAT interventions and/or bargaining. Results of effects on shares of energy and energy adequacy (in-takes/requirements) are similar.

8 We use the Goldberg method to define outliers, where individuals are outliers if the ratio between energy intakes and basal metabolic rate is < 1.16 (women) or < 1.19 (men). Basal metabolic rate is calculated using the Schofield equation and is based on age, gender, body weight.

9 MUAC, originally developed as a screening tool for identifying children with elevated risk of death, is increasingly used as a measure of nutritional status in adults and in pregnancy. In our case, it is preferable to other measures such as body-mass index or weight because it is less affected by pregnancy and therefore facilitates better comparison of individuals within households.

10 Wealth score was derived as the first principal component from a principal components analysis of binary variables indicating household ownership of 14 assets: improved toilet, improved water source, modern roof, modern floor, electricity access, colour television, motorbike, bicycle, sewing machine, ox cart, fridge, camera, computer, land.

11 Although there are physiological sex differences in body composition, analysis from Nepal show that the same MUAC cut-offs can be used for classifying underweight men and women. Thorup L, Hamann SA, Kallestrup P, Hjortdal VE, Tripathee A, Neupane D, Patsche CB. Mid-upper arm circumference as an indicator of underweight in adults: a cross-sectional study from Nepal. BMC public health 2020; 20; 1–7.

12 Analyses with the control arm indicate that households respond equally to the labour contributions of mothers-in-law and household heads, allocating 220 kcal/d (SE 70 and 73 respectively) more for strenuous v moderate physical activity, after adjusting for total household energy consumption. We find no effects of physical activity on intakes for daughters-in-law.

13 In absolute terms, total daily consumption of animal-source foods was 119 g higher and consumption of staple foods was 340 g lower than comparison group.

14 Compared with the lowest wealth tertile, household shares of staple foods (defined as grams of staples/grams of total food) are 3.7 pp lower in the middle wealth tertile ((95% CI −6.1 to –1.3), *p* = 0.002) and 9.1 pp lower the top tertile (−12.1 to −6.1, *p <* 0.001). Household shares of fruit and vegetable do not differ in the middle wealth tertile but are 2 pp higher in the top tertile compared with the lowest tertile ((95% CI 0.1 to 4.3), *p =* 0.042). Shares of animal-source foods are 2 pp higher in the middle tertile (95% CI 0.13 to 4.0), *p =* 0.037)) and 6.4 pp higher in the top tertile ((95% CI 4.1 to 8.7), *p <* 0.001). These results are from univariable analyses of 150 households in the control arm, using tertiles of a wealth score described in Section 4.1 as the independent variable, with cluster-robust standard errors.

15 Association with staples is –0.03 [95% CI −0.06 to 0.01], *p =* 0.194 and animal-source foods is 0.01 [−0.03 to 0.04], *p =* 0.713.

16 The 2015 Earthquakes did not affect the plains area much, although many of the research team with family in the hills were personally affected. The political conflict resulted in closure of trade across the Nepal-India border, restricted travel, and closure of markets, banks, and other businesses in the study districts. Although most transfers and meetings were implemented as planned, these factors may have restricted the community action elements of the PLA component.
