# Supplementary material for: Relative power: Explaining the effects of food and cash transfers on allocative behaviour in rural Nepalese households
Source: J Dev Econ. Author manuscript; Available in PMC 2022 Jan 1. (PMC7612026; doi:10.1016/j.jdeveco.2021.102784)
Supplement: Supplementary file 1 [file EMS138941-supplement-Appendix.pdf]

Declaration of competing interest

None.

Data availability

Data will be made available on request.

Acknowledgements

We thank Christopher Udry, Suneetha Kadiyala, and Lu Gram for their invaluable comments on earlier versions, and to Anthony Costello for supervision in earlier phases of this project. Thanks to Tom Harrison, Niva Shrestha, Sonali Jha, and B James Beard for their help with the development of the dietary assessment tools. We thank field supervisors, Devlal Bishwakarma, Shankar Kaphle, Suresh Mandal, and Dharambir Pasman. We thank the rest of the team involved in the Low Birth Weight South Asia Trial from Mother and Infant Research Activities (MIRA) (in particular Bhim Prasad Shrestha, Aman Sen, Sonali Anjana Rai, Bishnu Bhandari and Rishi Neupane, 66 data collectors, 16 supervisors, and 720 enumerators) and University College London (Sarah Style, Andrew Copas, Joanna Morrison, and Jayne Harthan).

Appendix A. CONSORT checklist

Section/Topic	Item No	Checklist item	Location or response
Title and abstract			
	1a	Identification as a randomised trial in the title	Provided in abstract
	1 b	Structured summary of trial design, methods, results, and conclusions	Provided in abstract
Introduction			
Background and objectives	2a	Scientific background and explanation of rationale	Provided in Sections 1 and 2
	2 b	Specific objectives or hypotheses	Provided in Section 2
Methods			
Trial design	3a	Description of trial design (such as parallel, factorial) including allocation ratio	Parallel, four-arm, cluster-randomised trial, allocation ratio 1:1:1:1
	3 b	Important changes to methods after trial commencement (such as eligibility criteria), with reasons	None for this sub-study
Participants	4a	Eligibility criteria for participants	Eligibility of clusters: Maithili-speaking clusters in Dhanusha or Mahottari districts, with no large towns, not on the East-West Highway, and not hilly or forested.<br>Eligibility for menstrual monitoring: Married women aged 10–49 years, who had not had tubal ligation and whose husbands had not had vasectomy.<br>Eligibility for interventions: Women with a positive pregnancy test or obviously pregnant appearance.<br>Eligibility for intra-household sub-study: Male-headed, joint households of permanently resident (enrolled in census or newly-wed in-migrating) women in their third trimester enrolled in the trial.
	4 b	Settings and locations where the data were collected	Setting: Dhanusha and Mahottari districts in floodplains of Nepal.<br>Location of data collection: Respondents' homes.
Interventions	5	The interventions for each group with sufficient details to allow replication, including how and when they were actually administered	Summarised in Section 2.<br>Described in full in Saville et al. (2018).
Outcomes	6a	Completely defined pre-specified primary and secondary outcome measures, including how and when they were assessed	Food shares calculated as intakes of daughter-in-law as a proportion of (i) daughter-in-law + mother-in-law, and (ii) daughter-in-law + male household head. Foods were staple foods, fruit and vegetables, and animal source foods. Other outcomes reported are shares of dietary diversity (a count of 10 food groups as defined by FAO (2014)) and mid-upper arm circumference (cm). This is a secondary analysis and outcomes were not pre-specified.
	6 b	Any changes to trial outcomes after the trial commenced, with reasons	N/A
Sample size	7a	How sample size was determined	Section 3.1. Target sample size was calculated as 200 per arm, to detect a two-sided difference in energy allocation ratios from 0.9 to 1.0 (assuming 0.27 SD and intra-cluster correlation of 0.03), with 80% power and a type I probability of 5%.
	7 b	When applicable, explanation of any interim analyses and stopping guidelines	N/A
Randomisation:			
Sequence generation	8a	Method used to generate the random allocation sequence	Block randomisation using a 'tombola method' with community stakeholders.
	8 b	Type of randomisation; details of any restriction (such as blocking and block size)	Four strata based on population size (4000–6399 vs. 6400–9200) and high or low accessibility during monsoon season.
Allocation concealment mechanism	9	Mechanism used to implement the random allocation sequence (such as sequentially numbered containers), describing any steps taken to conceal the sequence until interventions were assigned	Concealment of allocation was impossible due to the cluster-level study design.
Implementation	10	Who generated the random allocation sequence, who enrolled participants, and who assigned participants to interventions	Community stakeholders used the tombola to allocate clusters. Study enumerators monitored menstruation and enrolled women into the trial.
Blinding	11a		N/A

(continued on next page)

(*continued*)

Section/Topic	Item No	Checklist item	Location or response
		If done, who was blinded after assignment to interventions (for example, participants, care providers, those assessing outcomes) and how	
	11 b	If relevant, description of the similarity of interventions	N/A
Statistical methods	12a	Statistical methods used to compare groups for primary and secondary outcomes	We estimate intent-to-treat effects of the food and cash transfers on daughter-in-law's food shares relative to their mother-in-law and male household head by fitting multilevel linear regression models using maximum likelihood. We treat clusters as random effects. We report cluster robust standard errors, which are clustered at the VDC level.
	12 b	Methods for additional analyses, such as subgroup analyses and adjusted analyses	Described in Section 3.4 and 3.5.
Results			
Participant flow (a diagram is strongly recommended)	13a	For each group, the numbers of participants who were randomly assigned, received intended treatment, and were analysed for the primary outcome	We interviewed 805/1074 (75%) eligible households, and include 800 in our analytical sample.
	13 b	For each group, losses and exclusions after randomisation, together with reasons	Reasons for attrition were migration (n = 13), respondents not available (n = 219), unable to locate home (n = 1), declined to consent (n = 23), and no reason reported (n = 13). Of 805 interviewed households, we exclude 5 due to missing demographic data.<br>Arm-wise attrition given in Harris-Fry et al. (2018).
Recruitment	14a	Dates defining the periods of recruitment and follow-up	Trial enrolment between Dec 2013 and Feb 2015.<br>Dietary measurements between May and Sep 2015.
	14 b	Why the trial ended or was stopped	Low capture of primary outcome (birthweight), exacerbated by ethnic conflict in field team and lack of funds to continue the study.
Baseline data	15	A table showing baseline demographic and clinical characteristics for each group	Table 1
Numbers analysed	16	For each group, number of participants (denominator) included in each analysis and whether the analysis was by original assigned groups	Table 1
Outcomes and estimation	17a	For each primary and secondary outcome, results for each group, and the estimated effect size and its precision (such as 95% confidence interval)	Intent-to-treat results in Tables 3 & 4
	17 b	For binary outcomes, presentation of both absolute and relative effect sizes is recommended	N/A
Ancillary analyses	18	Results of any other analyses performed, including subgroup analyses and adjusted analyses, distinguishing pre-specified from exploratory	Further analyses in Fig. 2, Tables 5 and 6<br>All analyses are exploratory.
Harms	19	All important harms or unintended effects in each group (for specific guidance see CONSORT for harms)	Potential harm in terms of nutrition of mothers-in-law is discussed. Monitoring of harms reported in Saville et al. (2018).
Discussion			
Limitations	20	Trial limitations, addressing sources of potential bias, imprecision, and, if relevant, multiplicity of analyses	Limitations discussed in Section 5.<br>Limitations of mediation analyses discussed in Section 3.5
Generalisability	21	Generalisability (external validity, applicability) of the trial findings	Section 5.
Interpretation	22	Interpretation consistent with results, balancing benefits and harms, and considering other relevant evidence	Sections 4 & 5.
Other information			
Registration	23	Registration number and name of trial registry	ISRCTN 75964374
Protocol	24	Where the full trial protocol can be accessed, if available	Saville et al. (2018)
Funding	25	Sources of funding and other support (such as supply of drugs), role of funders	Main trial funder: UK Department for International Development (DFID; grant PO 5675).<br>Funding of author HHF: Child Health Research Charitable Incorporated Organisation, and Wellcome Trust Grant Award Number: 210894/Z/18/Z.<br>Role: Funders had no role in study design; collection, analysis, or interpretation of data; writing of the report; or decision to submit the article for publication.

Appendix B. Prediction of usual intakes

For occasionally consumed foods that have a truncated distribution (animal-source foods), we predict the conditional mean intake by fitting a two-part model with person-specific random effects, where the probability of consumption is estimated using a multilevel logistic regression, the amount consumed on consumption days is estimated by fitting a multilevel nonlinear regression model, and the error terms of the two parts are correlated. Usual intakes of individual nutrients (energy, iron, vitamin A) and the other foods (staples, fruit and vegetables) are consumed on most days so are estimated using only the 'amount part' (the nonlinear regression model).

This approach follows validated, standard methods developed by National Cancer Institute (NCI) to deal with the wide within-person variance of ubiquitously or episodically consumed foods, and is required to address the attenuation of associations between intakes and covariates that would arise if using a simple person-specific mean intake (Dodd et al., 2006; Kipnis et al., 2009; Tooze et al., 2010).

Using subindices $_1$ and $_2$ to denote the first and second parts of the model, the consumption probability of a food or nutrient F for an individual v on day w*,* is estimated in (i) as:

$$\Pr\left(F_{vw} > 0|v\right) = \alpha_{10} + \alpha_{1b}b_v + \alpha_{1t}t_v + \alpha_{1X}X_v + u_{1v},\ w = 1, \dots, W_v \tag{A1}$$

and intake of F on consumption days is predicted as:

$$F_{vw} = \alpha_{20} + \alpha_{2b}b_v + \alpha_{2t}t_v + \alpha_{2x}X_v + u_{2v} + \varepsilon_{2vw}, \tag{A2}$$

where b is a measure of bargaining power, t indicates trial arm, $\boldsymbol{X}$ is a vector of other household characteristics (randomisation stratum and whether the household head is the daughter-in-law's husband), and u_j and ε_{2vw} denote normally distributed within-person effects and person-specific error terms respectively.

Table A1
Sample attrition

		Means or proportions for participants who were eligible but not sampled, $n = 269$				p-value of equality			
						Control vs			PLA + food vs
	N	Control	PLA	PLA + cash	PLA + food	PLA	PLA + cash	PLA + food	PLA + cash
Attrited proportion		0.25	0.28	0.22	0.27	0.43	0.69	0.82	0.48
Muslim or Dalit	266	0.48	0.42	0.33	0.35	0.53	0.16	0.20	0.83
Household asset score	260	−0.27	−0.27	−0.06	−0.18	0.99	0.54	0.76	0.61
Household size	266	6.59	6.77	6.81	7.09	0.81	0.77	0.50	0.70
Age, daughter-in-law	266	22.2	23.0	22.6	22.9	0.38	0.61	0.44	0.68
Education, years, wife	260	2.22	2.31	3.54	2.45	0.91	0.10	0.74	0.06
Education, years, husband	259	3.24	4.10	4.28	3.72	0.28	0.24	0.54	0.55

Note: Test for equality between arms based on cluster-robust standard errors.
Household asset score = First principal component from 14 assets owned by household: improved toilet, improved water source, modern roof, modern floor, electricity access, colour television, motorbike, bicycle, sewing machine, ox cart, fridge, camera, computer, land. Some missing data on attrited sample missing because it was not collected in the main trial's surveillance system.

Table A2
Intent-to-treat estimates of the effect of food and cash transfer interventions on food shares – unadjusted results

	PLA + cash vs. Control & PLA		PLA + food vs. Control & PLA	
	Mean difference [95% CI]	*p*-value	Mean difference [95% CI]	*p*-value
Shares between daughters-in-law and mothers-in-law				
Staples	1.91	0.011	2.16	<0.001
	[0.43, 3.38]		[0.98, 3.34]	
Fruit & veg	1.73	0.018	0.16	0.850
	[0.29, 3.16]		[-1.54, 1.87]	
Animal-source foods	1.86	0.080	1.23	0.337
	[-0.22, 3.96]		[-1.27, 3.73]	
Shares between daughters-in-law and male household heads				
Staples	0.33	0.617	1.36	0.089
	[-0.97, 1.63]		[-0.21, 2.93]	
Fruit & veg	0.68	0.378	0.11	0.891
	[-0.74, 1.94]		[-1.41, 1.62]	
Animal-source foods	1.43	0.019	2.12	0.172
	[0.55, 6.15]		[-0.92, 5.16]	
N	582		519	

95% CIs based on cluster-robust SEs. Models adjust for clustered and stratified study design only.

Table A3
Effect of PLA + cash and PLA + food interventions on allocation of mid-upper arm circumference and dietary diversity

	PLA + cash vs. Control & PLA		PLA + food v. Control & PLA	
**Shares between daughters-in-law and mothers-in-law**				
Adjusted model	$n = 582$			
Mid-upper arm circumference	0.65	0.026	0.20	0.593
	[0.08, 1.23]		[-0.53, 0.92]	
Diet diversity	0.36	0.570	1.04	0.190
	[-0.88, 1.59]		[-0.52, 2.61]	
Unadjusted model	*n = 587*			
Mid-upper arm circumference	0.63	0.045	0.23	0.526
	[0.01, 1.25]		[-0.48, 0.95]	
Diet diversity	0.38	0.553	1.02	0.200
	[-0.88, 1.65]		[-0.54, 2.58]	
**Shares between daughters-in-law and male household heads**				
Adjusted model	$n = 582$			
Mid-upper arm circumference	0.26	0.404	0.40	0.106
	[-0.35, 0.86]		[-0.09, 0.89]	
Diet diversity	1.72	0.014	2.09	0.001

(*continued on next page*)

Table A3 (*continued*)

	PLA + cash vs. Control & PLA		PLA + food v. Control & PLA	
	[0.35, 3.08]		[0.87, 3.32]	
Unadjusted model	*n* = *587*			
Mid-upper arm circumference	0.33	0.269	0.46	0.063
	[-0.25, 0.91]		[-0.02, 0.95]	
Diet diversity	1.77	0.008	2.22	<0.001
	[0.46, 3.09]		[1.03, 3.42]	

Note: 95% CIs based on cluster-robust SEs. Unadjusted models adjust for clustered and stratified study design only. Adjusted models adjust for clustered and stratified study design, plus controls for caste group, wealth, women's education, and season.

Table A4
Average marginal effects of food and cash transfers on power share when husband works overseas

	Moderator	*n*	Effect	95% CI	*p*-value for interaction
Treatment: PLA + cash					
Adjusted model					
Average treatment effect		573	4.89	[0.97, 8.80]	
Average marginal effect	Husband working overseas	93	−1.99	[-9.30, 5.31]	0.040
	Husband not overseas	480	6.12	[2.07, 10.17]	
Unadjusted model					
Average treatment effect		573	5.89	[1.90, 9.87]	
Average marginal effect	Husband working overseas	93	−2.21	[-9.46, 5.05]	0.037
	Husband not overseas	480	5.89	[1.90, 9.87]	
Treatment: PLA + food					
Adjusted model					
Average treatment effect		512	0.27	[-2.68.3.22]	
Average marginal effect	Husband working overseas	98	−3.80	[-12.08, 4.49]	0.260
	Husband not overseas	414	1.15	[-1.77, 4.08]	
Unadjusted model					
Average treatment effect		512	1.18	[-1.69, 4.05]	
Average marginal effect	Husband working overseas	98	−3.45	[-12.1, 5.20]	0.307
	Husband not overseas	414	1.18	[-1.69, 4.05]	

Note: Adjusted model controls: caste group, wealth score, daughter-in-law's education, household size and study stratum. Unadjusted results control for clustering and study strata only. Average treatment effects are slightly different to those reported in Table 4 because we are missing data on overseas migration in 11 households. 95% CIs based on cluster-robust SEs.

Table A5
Sensitivity analyses showing ρ at which ACME = 0

Exposure	Mediator	Outcome	ρ
PLA + cash	Power score	Allocation of fruit and veg between DIL & MIL	0.10
		Allocation of animal-source foods between DIL & HHH	0.10
PLA + cash	Power share	Allocation of staples between DIL & MIL	0.13
		Allocation of fruit and veg between DIL & MIL	0.10

Note: ρ denotes correlation between error terms of the mediator and outcome. DIL = daughter-in-law; HHH = household head; MIL = mother-in-law

References

Agarwal, B., 1994. A Field of One's Own: Gender and Land Rights in South Asia. Cambridge University Press.

Ahmed, A., Hoddinott, J., Roy, S., 2019. Food Transfers, Cash Transfers, Behavior Change Communication and Child Nutrition: Evidence from Bangladesh. Intl Food Policy Res Inst.

Ahmed AU, Quisumbing AR, Hoddinott JF, Nasreen M, Bryan E. Relative efficacy of food and cash transfers in improving food security and livelihoods of the ultra-poor in Bangladesh. International Food Policy Research Institute, in collaboration with Dhaka University, American University, and Data Analysis and Technical Assistance Limited, http://documents. wfp. org/stellent/groups/public/documents/liaison_ offices/wfp144615. pdf 2007.

Akerele, D., 2011. Intra-household food distribution patterns and calorie inadequacy in South-Western Nigeria. Int. J. Consum. Stud. 35, 545–551.

Almås, I., Armand, A., Attanasio, O., Carneiro, P., 2018. Measuring and changing control: women's empowerment and targeted transfers. Econ. J. 128, F609–F639.

Ambler, K., De Brauw, A., 2017. The Impacts of Cash Transfers on Women's Empowerment: Learning from Pakistan's BISP Program. World Bank.

Anukriti, S., Herrera-Almanza, C., Karra, M., Pathak, P., 2020. Curse of the mummy-ji: the influence of mothers-in-law on women's social networks, mobility, and reproductive health in India. Am. J. Agric. Econ.

Armand, A., Attanasio, O., Carneiro, P.M., Lechene, V., 2016. The Effect of Gender-Targeted Conditional Cash Transfers on Household Expenditures: Evidence from a Randomized Experiment.

Attanasio, O.P., Lechene, V., 2014. Efficient responses to targeted cash transfers. J. Polit. Econ. 122, 178–222.

Aubel, J., 2012. The role and influence of grandmothers on child nutrition: culturally designated advisors and caregivers. Matern. Child Nutr. 8, 19–35.

Babu, S.C., Thirumaran, S., Mohanam, T., 1993. Agricultural productivity, seasonality and gender bias in rural nutrition: empirical evidence from South India. Soc. Sci. Med. 37, 1313–1319.

Bastagli, F., Hagen-Zanker, J., Harman, L., Barca, V., Sturge, G., Schmidt, T., Pellerano, L., 2016. Cash Transfers: what Does the Evidence Say? A Rigorous Review of Programme Impact and of the Role of Design and Implementation Features. Overseas Development Institute, London, UK. URL: https://www.odi.org/sites/odi.org.uk/files/resource-documents/10749.pdf. (Accessed 27 October 2016).

Becker, G.S., 1981. A Treatise on the Family. Harvard University Press.

Behrman, J.R., 1988. Nutrition and Incomes: Tightly Wedded or Loosely Meshed? Pew. Cornell Lecture Series on Food and Nutrition Policy-Cornell University (EUA).

Behrman, J.R., Deolalikar, A.B., 1990. The intrahousehold demand for nutrients in rural south India: individual estimates, fixed effects, and permanent income. J. Hum. Resour. 665–696.

Bennett, L., 1983. Dangerous Wives and Sacred Sisters. Social and Symbolic Roles of High Caste Women in Nepal. Columbia University Press, New York.

Berti, P.R., 2012. Intrahousehold distribution of food: a review of the literature and discussion of the implications for food fortification programs. Food Nutr. Bull. 33.
